# Consecutive first-morning urine samples to measure change in the albumin-to-creatinine ratio: a pilot study of a home urine collection protocol

**DOI:** 10.1186/s40697-016-0095-8

**Published:** 2016-02-01

**Authors:** Jessica M. Sontrop, Amit X. Garg, Lihua Li, Kerri Gallo, Virginia Schumann, Jennifer Winick-Ng, William F. Clark, Matthew A. Weir

**Affiliations:** Department of Epidemiology and Biostatistics, Western University, London, ON N6A 5C1 Canada; Division of Nephrology, Department of Medicine, London Health Sciences Centre, London, ON N6A 4G5 Canada

**Keywords:** ACR, Albumin-to-creatinine ratio, Chronic kidney disease, First-morning urine samples, Pilot study

## Abstract

**Background:**

Multiple first-morning urine samples are recommended for measuring the urine albumin-to-creatinine ratio (ACR); however, this can be challenging in community-based research.

**Methods:**

The objectives of the study are to pilot-test a home urine collection protocol and examine how the average and variance of ACR varied with the number of urine collections and time to laboratory analysis. This is a prospective observational pilot study. This study was conducted in London, Ontario, Canada at the London Health Sciences Centre (2012–2013). The patients were adults with chronic kidney disease (mean estimated glomerular filtration rate, 36 mL/min/1.73 m^2^). Participants collected a first-morning 20-mL urine sample on three consecutive days. This process was repeated after 3 months. Samples were picked up by hospital courier and analyzed for ACR on the same day; additional aliquots were analyzed after a delay of 24–48 h (stored at 4 °C) and 3–9 months (stored at –80 °C). The geometric mean of the percentage change in ACR between baseline and 3 months was calculated and compared between single samples and the average of two vs. three consecutive samples.

**Results:**

Of 31 patients enrolled, 26 (83.9 %) submitted all six urine samples. The geometric mean of ACR for three consecutive samples at baseline was 87, 83, and 80 mg/mmol, and the corresponding percentage increase from baseline to 3 months was 15 % (95 % confidence interval (CI), −9 to 46 %), 33 % (95 % CI, 10 to 59 %), and 22 % (95 % CI, −6 to 57 %). Compared with single urine collections at baseline and follow-up, averaging ACR values from two consecutive first-morning urine samples improved the sample variance and reduced the required sample size to detect a given treatment effect by approximately 30 %. No further gain in statistical efficiency was achieved with three urine samples. Results were similar when the laboratory analysis was delayed by 24–48 h, but a delay of 3–9 months resulted in systematic overestimation of the ACR. Our study’s generalizability is limited by its small sample size and reliance on a clinic-based population from a single urban center.

**Conclusions:**

We successfully used a home urine collection protocol to obtain multiple first-morning urine samples in patients with chronic kidney disease. Statistical efficiency was improved by averaging ACR values from two consecutive first-morning urine samples at baseline and follow-up.

## ABRÉGÉ

**Mise en contexte:**

Le prélèvement de la première urine du matin est recommandé pour procéder à l’analyse du ratio albumine/créatinine (RAC) chez les patients atteints d’insuffisance rénale chronique (IRC). Toutefois, cette procédure représente un défi dans le domaine de la recherche communautaire.

**Objectifs de l’étude:**

Cette étude constitue un essai pilote qui visait à établir un protocole normalisé pour la collecte d’échantillons d’urine chez les patients atteints d’IRC. Cet essai pilote avait également pour objectif de caractériser les variations dans les valeurs moyennes et les valeurs d’écart à la moyenne des RAC en fonction du nombre de prélèvements effectués et du moment où ceux-ci sont analysés en laboratoire.

**Cadre et type d’étude:**

Il s’agit d’une étude pilote prospective réalisée par observation. L’étude s’est tenue au *London Health Science Center* de London en Ontario, au Canada en 2012 et 2013.

**Méthode:**

Les participants, des adultes atteints d’IRC (débit de filtration glomérulaire moyen de 36 mL/min/1,73 m^2^), ont procédé au prélèvement de 20 mL de la première urine du matin durant trois jours consécutifs. La procédure a été répétée après trois mois. À chaque fois, les échantillons étaient cueillis par un messager envoyé par l’hôpital pour leur analyse du RAC le jour-même. De chacun des échantillons reçus, deux aliquots étaient prélevés pour analyse ultérieure: un premier était conservé à 4 °C pour analyse dans les 24 à 48 heures, et un deuxième était congelé à −80 °C pour analyse du RAC de 3 à 9 mois suivant le prélèvement. La moyenne géométrique des pourcentages de variation du RAC entre les valeurs mesurées le jour-même et les valeurs mesurées après 3 mois a été calculée, on l’a ensuite comparée avec le RAC des échantillons individuels avec les moyennes de RAC obtenues pour deux et trois échantillons consécutifs.

**Résultats:**

Des 31 patients inclus dans l’étude, 83,9 % (n = 26) ont fourni les six échantillons d’urine requis pour analyse. Les moyennes géométriques des RAC pour les échantillons prélevés sur trois jours consécutifs s’élevaient à 87,83 et 80 mg/mmol, et les pourcentages correspondants à la variation entre le RAC au jour 1 et le RAC après 3 mois étaient respectivement de 15 % (95 % IC : −9 % à 46 %), de 33 % (95 % IC : 10 % à 59 %), et de 22 % (95 % IC : −6 % à 57 %). En comparant les RAC des échantillons individuels au jour 1 et après 3 mois avec la moyenne des RAC de deux échantillons prélevés consécutivement, un accroissement de la variance a été observé. De plus, cette comparaison a également permis de conclure qu’il était possible de détecter un effet thérapeutique donné dans un échantillon jusqu’à 30 % moins volumineux. Aucun avantage sur le plan de l’efficacité statistique n’a été amené par l’analyse de trois prélèvements consécutifs. Enfin, les résultats d’analyse du RAC de 24 à 48 heures suivant l’arrivée des échantillons n’a offert que peu de variation en comparaison avec les valeurs obtenues au jour 1. Par contre, dans le cas des échantillons analysés après 3 à 9 mois ceux-ci ont systématiquement entraîné une surévaluation de la mesure du RAC.

**Limites de l’étude:**

La possibilité de généralisation de la présente étude est limitée par son faible échantillonnage et par le fait qu’elle s’appuie sur une population clinique provenant d’une seule agglomération urbaine.

**Conclusions:**

L’essai pilote mené dans le but d’établir un protocole normalisé permettant aux patients atteints d’IRC de prélever des échantillons d’urine du matin à la maison a été couronné de succès. De plus, les résultats montrent que d’utiliser les moyennes de RAC de deux échantillons prélevés consécutivement permet d’accroître l’efficacité statistique tant pour les échantillons analysés au jour 1 que lors du suivi après 3 mois.

## What was known before

Measurement of the albumin-to-creatinine ratio is hindered by substantial intra-individual variation. Obtaining multiple consecutive first-morning urine samples can improve the precision of this measure; however, because samples must be kept at 4 °C, obtaining first-morning samples from outpatients can be challenging.

## What this study adds

We used a home urine collection protocol to obtain first-morning urine samples in outpatients with chronic kidney disease (26 of 31 patients submitted all six urine samples: three at baseline and three at 3-month follow-up). Compared to single urine collections, averaging the albumin-to-creatinine ratio from two consecutive first-morning urine samples improved the sample variance and reduced the required sample size to detect a given treatment effect by approximately 30 %.

## Background

To determine whether an intervention can prevent end-stage kidney disease or mortality, large multicenter trials with thousands of patients are typically required. However, before such trials can proceed, supporting evidence using intermediate outcomes such as albuminuria is needed. A rise in the level of albuminuria is an early indicator of kidney disease and an established risk factor for cardiovascular disease [1, 2]. The change in albuminuria, usually measured by the albumin-to-creatinine ratio (ACR) in a random urine sample, is a frequent surrogate endpoint in early-phase clinical trials testing new therapies to slow the progression of kidney disease [3–5].

The precision of ACR measurement is reduced by substantial intra-individual variation related to diurnal and biological factors, diet and exercise, and also by factors related to sample storage and processing [6–8]. High variability in this outcome measure could potentially obscure a beneficial intervention effect or require larger sample sizes to see a given treatment effect, and this could substantially increase a study’s costs. One solution to this problem is to obtain multiple consecutive first-morning urine samples (the first urine void after waking from sleep); ACR measured from first-morning urine samples has been shown to be comparable to the gold standard 24-h urine collection, with better precision and less intra-individual variability than random spot urine samples (which are usually collected at a clinic visit) [7–12]. Ideally, first-morning urine samples should be kept at 2–8 °C (to minimize the degradation of ACR which occurs at higher temperatures) [8]; however, this can make it challenging to obtain first-morning urine samples from outpatients in community-based research. To address this issue, we designed a home urine collection protocol modeled after a protocol developed by the National Health and Nutrition Examination Survey [13, 14]. Patients were instructed to collect first-morning urine samples at home (on three consecutive days), and samples were picked up by hospital courier for same-day analysis of ACR. The purpose of this study was to pilot-test this protocol to guide optimal albuminuria measurement in future randomized controlled trials. We examined how the average and variance of ACR varied with the number of urine collections and time to laboratory analysis.

## Methods

Adult patients (age 18–80 years) attending a Chronic Kidney Disease Clinic at Victoria Hospital in London, ON (Canada 2012–2013) who met the study’s eligibility criteria were invited to participate. We included patients whose most recent estimated glomerular filtration rate (eGFR) was between 20 and 60 ml/min/1.73 m^2^ and whose random urine ACR exceeded 30 mg/mmol. We excluded patients who met any of the following criteria: residence outside London, ON; enrollment in another study that could influence the data collection of this study; receipt of one or more dialysis treatments in the past month; kidney transplant recipient (or on waiting list); pregnant or breastfeeding; life expectancy less than 6 months; recent diagnosis (<6 months) with vasculitis, proliferative glomerulonephritis, or lupus nephritis or those who received intravenous cyclophosphamide in the previous 6 months; and those diagnosed with primary nephritic syndromes. The patient’s healthcare provider introduced the study and interested patients spoke to the study’s research assistant who explained the study, confirmed eligibility, and obtained written informed consent. Ethics approval was obtained from Western University’s Health Sciences Research Ethics Board. We planned to enroll 30 patients as recommended by Julious and Moore et al. for preparatory pilot studies assessing feasibility and the estimation of continuous outcome variables for planning larger subsequent studies [15, 16].

### Home urine collection protocol

We created a home urine collection (HUC) protocol that was modeled after a protocol developed by the National Health and Nutrition Examination Survey [13]. The research assistant gave each participant a re-useable carrying bag containing three pre-assembled HUC kits and a packet of detailed instructions for collection, storage, and delivery of the samples. The research assistant provided verbal, written, and visual instructions to each participant.

We asked participants to collect a 20-mL urine sample first thing in the morning for three consecutive days at baseline (Monday, Tuesday, and Wednesday) and again 3 months later. We provided patients with biohazard bags containing absorbent pads and instructed them to place their urine sample in the bag immediately after collection and seal it closed. Bags were placed in an insulated shipping container with a frozen ice pack (placed in freezer the night before). We arranged for a certified hospital courier to pick up each sample on the same morning of collection. Samples were delivered to the London Health Sciences Centre (LHSC) for processing. Each 20-mL sample was divided into multiple 1.0-mL aliquots: one aliquot was analyzed immediately (on the same day) for ACR, two aliquots were stored at 4 °C for delayed analysis at 24 and 48 h, and the fourth aliquot was stored at −80 °C for batched analysis at the study’s end, approximately 3 to 9 months after recruitment was completed. This process was repeated again 3 months after baseline. Blood samples were collected at both clinic visits and analyzed for serum creatinine. eGFR was calculated using the Chronic Kidney Disease Epidemiology equation [17]. The urine albumin-to-creatinine ratio (ACR) was analyzed using an immunoturbidimetric inhibition assay with a lower limit of detection 0.05 mg/L. All samples were analyzed at the same laboratory (London Health Sciences Centre Core Labs) on one of two Roche/Hitachi machines with the same analyzer (the COBAS INTEGRA 800) using the Tina-quant Albumin Gen.2 reagent). Samples were centrifuged prior to analysis for 10 min at ≥800 g.

### Statistical analysis

We summarized normally distributed data using means and standard deviations (SD) and non-normally distributed data using medians and interquartile ranges (IQR). We calculated the percentage change in ACR (month 3/baseline) for single samples and for the average of two and three consecutive samples. Because the distribution of ACR is skewed, we transformed these data using the natural logarithm and present the geometric mean of the percentage change (geometric mean, 1) × 100 %; 95 % confidence intervals (95 % confidence interval (CI)) were calculated using a *t* value of 2.060 (*t*_0.975_ with 25 degrees of freedom). Our primary statistical analysis used urine ACR values obtained from the same-day laboratory analysis. To examine the effect of delayed laboratory analysis, we compared mean ACR measured on the same day of urine collection with the mean ACR measured after a delay of 24 h (stored at 4 °C), 48 h (stored at 4 °C), and 3–9 months (stored at −80 °C and analyzed in one batch). Differences in mean ACR were compared using paired *t* tests of the log-transformed data.

We examined the effect of averaging multiple ACR tests on sample size requirements using formulae for log-transformed data as described in Wolfe and Carlin [18]. For these calculations, we used the standard deviation of the observed log-transformed percentage change in ACR in this study and specified between-group differences in the geometric mean of the percentage change in ACR ranging from 10 to 20 % (most physicians would view a 20 % reduction in albuminuria as evidence of a promising treatment effect; such was the case for ACE inhibitors and angiotensin receptor blockers, which were later proven to reduce the risk of mortality and end-stage renal disease) [4, 19–21]. All analyses were performed using SAS software version 9.3 (SAS Institute Inc., Cary, NC, USA).

## Results

We enrolled 31 patients with chronic kidney disease between June 5, 2012 and December 11, 2012. Of 31 participants, 26 (83.9 %) collected all three consecutive first-morning urine samples at both baseline and 3-month follow-up and 30 (96.8 %) collected at least two first-morning urine samples at each of these time points. Characteristics of the 26 participants who provided all six urine samples are summarized in Table 1: 89 % were men and 77 % Caucasian, and the average age was 63 years (SD 14); 89 % had hypertension and 69 % had diabetes. Average eGFR at baseline was 36 ml/min/1.73 m^2^ (SD 15).

**Table 1:** Sample characteristics at baseline (*n* = 26)

Mean age, years (SD) [min-max]	62.9 (14.0) [31–84]
Men, *n* (%)	23 (88.5 %)
Caucasian, *n* (%)	20 (76.9 %)
Mean body mass index, kg/m^2^ (SD) [min-max]	32 (6) [25–45]
Mean serum creatinine, umol/L (SD) [min-max]	197 (74) [104–349]
Mean eGFR, ml/min/1.73 m^2^ (SD) [min-max]	36 (15) [15–60]
Mean SBP, mm Hg (SD) [min-max]	140 (25) [108–192]
Mean DBP, mm Hg (SD) [min-max]	75 (10) [50–97]
History of smoking, *n* (%)	15 (57.7 %)
Current smoker, *n* (%)	3 (11.5 %)
Primary CKD diagnosis, *n* (%)	
Diabetes	15 (57.7 %)
Hypertension	2 (7.7 %)
Interstitial nephritis	3 (11.5 %)
Other	6 (23.1 %)
Comorbidities, *n* (%)	
Hypertension	23 (88.5 %)
Hyperlipidemia	23 (88.5 %)
Diabetes	18 (69.2 %)
Coronary artery disease	6 (23.1 %)
Chronic obstructive pulmonary disorder	5 (19.2 %)
CVA/TIA	4 (15.4 %)
Peripheral vascular disease	1 (3.8 %)
Congestive heart failure	2 (7.7 %)
Gastric bleeding	1 (3.8 %)

*CKD* chronic kidney disease, *CVA/TIA* cerebrovascular accident/transient ischemic attack, *eGFR* estimated glomerular filtration rate, *SD* standard deviation

At baseline, mean ACRs for first-morning urine collections on days 1, 2, and 3 were 87, 83, and 80 mg/mmol, respectively, and at 3 months were 101, 110, and 97 mg/mmol. Summary statistics are shown in Tables 2 and 3. For single samples, the percentage change in ACR ranged 15 % (95 % CI, −9 to 46 %) to 33 % (95 % CI, 10 to 59 %). For the average of two consecutive samples, the percentage change in ACR was 24 % (95 % CI, 2 to 51 %), and for the average of three consecutive samples, the percentage change in ACR was 23 % (95 % CI, 0 to 50 %).

**Table 2:** Albumin-to-creatinine ratio (mg/mmol) from three consecutive first-morning urine samples collected at baseline and at 3-month follow-up (*n* = 26)

Sample collection	Geometric mean (95 % CI)	Median (interquartile range)	Min, max
Baseline			
Day 1	87 (59.1 to 128.7)	104.3 (41, 163)	15, 459
Day 2	83 (55.6 to 124.0)	98.7 (42, 159)	12, 453
Day 3	80 (52.3 to 121.0)	93.0 (35, 165)	11, 506
3 months			
Day 1	101 (66.7 to 151.4)	119.3 (40, 239)	15, 623
Day 2	110 (73.1 to 165.9)	107.1 (62, 257)	14, 729
Day 3	97 (65.5 to 143.1)	102.1 (39, 246)	15, 541

Samples were analyzed for the albumin-to-creatinine ratio on the same day of collection

**Table 3:** Change in the albumin-to-creatinine ratio between baseline and 3-month follow-up (*n* = 26)

Number of urine samples	Percentage change in ACR (geometric mean and 95 % CI)
One sample^a^	15.2 % (−9.2 to 46.1 %)
Average of two consecutive samples^b^	24.3 % (2.1 to 51.3 %)
Average of three consecutive samples^c^	22.6 % (0.0 to 50.3 %)

*ACR* albumin-to-creatinine ratio

^a^First urine sample

^b^First and second urine samples

^c^First, second, and third urine samples

### Effect of delayed laboratory analysis on ACR estimation

**Fig. 1:**
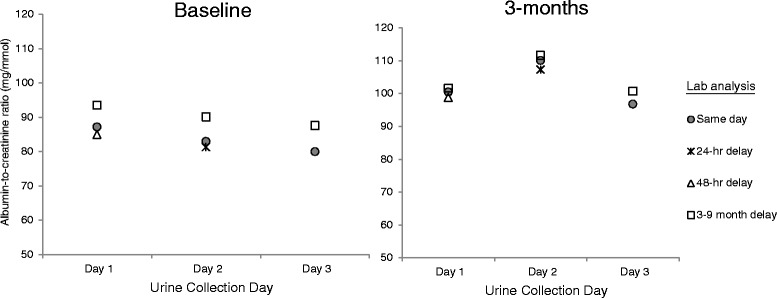
Effect of delayed laboratory analysis on the geometric mean of the urine albumin-to-creatinine ratio at baseline and 3 months (*n* = 26)

**Fig. 2:**
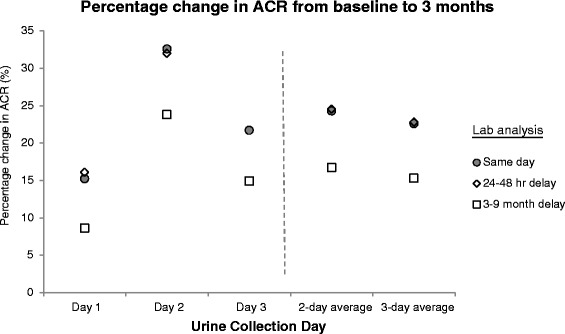
Effect of delayed laboratory analysis on the geometric mean of the percentage change in the urine albumin-to-creatinine ratio between baseline and 3 months (*n* = 26)

The effect of delayed laboratory analysis on ACR is summarized in Figs. 1 and 2. Compared with aliquots analyzed immediately, the mean ACR of aliquots refrigerated for a period of 24–48 h was not appreciably different; however, when the laboratory analysis was delayed by 3–9 months, the mean ACR was significantly higher by 6–8 mg/mmol. For example, for the same-day analysis of the first baseline urine collection, the mean ACR was 87 mg/mmol (95 % CI, 59 to 129). When the laboratory analysis of this sample was delayed by 48 h, the mean ACR was 85 mg/mmol (95 % CI, 58 to 125), and when delayed by 3–9 months, the mean ACR was 94 mg/mmol (95 % CI, 64 to 138). A corresponding decrease in the percentage change in ACR was evident when the laboratory analysis was delayed by 3–9 months, and these differences were statistically significant. The geometric means of the percentage change are shown in Fig. 2. For the same-day analysis of the day 1 urine collections at baseline and follow-up, the percentage change was 15.2 % (95 % CI, (−9.2 to 46.1 %) and this decreased to 8.6 % (95 % CI, (−14.1 to 37.2 %) when laboratory analysis was delayed by 3–9 months.

### Effect of averaging multiple ACR tests on variability and sample size requirements

Sample size calculations for various scenarios are summarized in Table 4. The standard deviation of the log-transformed percentage change in ACR was 0.59 for the first urine sample and 0.49 for the average of two consecutive samples; averaging ACR from three samples did not substantively reduce the standard deviation (0.50). Based on these data, detecting a between-group difference of at least 15 % in the geometric mean of the percentage change in ACR would require a total sample size of at least 414 participants (for single samples collected at baseline and follow-up); this number reduces to 286 if ACR from two consecutive first-morning urine samples is averaged (at both baseline and follow-up).

**Table 4:** Total sample size needed to detect a 10 to 20 % difference between two groups in the geometric mean of the percentage change in ACR under different scenarios

		Minimum detectable difference between groups		
Number of urine samples at each time point (baseline and follow-up)	Log-scale standard deviation^a^	10 %	15 %	20 %
One sample	0.59	984	414	220
Average of two consecutive samples	0.49	678	286	152
Average of three consecutive samples	0.50	706	298	158

Based on a type I error of 5 % and power of 80 %

^a^Standard deviation of the log-transformed percentage change in the albumin-to-creatinine ratio between baseline and 3-month follow-up

## Discussion

In this study, we successfully used a home urine collection protocol to obtain multiple first-morning urine samples in patients with chronic kidney disease. Of 31 participants, 84 % collected all three consecutive first-morning urine samples at both baseline and 3-month follow-up and 94 % collected at least two first-morning urine samples at each of these time points. We calculated the percentage change in ACR between baseline and 3-month follow-up and examined how the average and variance of ACR varied with the number of urine collections and time to laboratory analysis. The percentage change in ACR between baseline and 3 months ranged from 15 to 33 %. Averaging ACR values from two consecutive first-morning urine collections resulted in smaller standard deviations of the percentage change compared with single urine collections and reduced the required sample size by approximately 30 %. No further gain in statistical efficiency was achieved with three urine samples.

In a recent study that examined data on albuminuria from three clinical trials [22], Kropelin et al. found that the precision of anti-albuminuric drug effects could be improved by increasing the frequency of urine collections at both baseline and follow-up and, further, by incorporating additional albuminuria measurements in between these time points: in general, the greater the number of urine collections, the greater the precision and gain in statistical power. While these data demonstrate that repeated measurements for albuminuria can lower the sample size requirements for a trial, this gain in statistical efficiency has to be balanced against the increased burden to the participant and research personnel. For a trial with three consecutive first-morning urine collections, one method of reducing participant burden might be to store urine samples in the refrigerator until the third day when a hospital courier would pick up all three samples. For this reason, we examined the effect of delaying the laboratory analysis of urine samples for ACR by 24 to 48 h (stored at 4 °C) and observed no effect on the results. While promising, not all participants may be willing to store urine samples in their refrigerators, and this could be a potential deterrent to study participation. We found that delaying the laboratory analysis of samples by 24–48 h (stored at 4 °C) did not affect the results; however, delaying the analysis by 3–9 months (stored at −80 °C) resulted in a systematic overestimation of ACR of approximately 8 %. The latter observation contrasts to previous studies which found either no change in ACR [23–25] or a 10 % decrease after long-term storage of frozen samples [26–28]. While a detailed investigation into the effects of sample processing on ACR measurement was beyond the scope of this study, others have shown that laboratory assay parameters (which are not standardized across laboratories) can affect ACR measurement. These factors include freezing and thawing protocols, type of assay tube (glass, polystyrene, and polypropylene), mixing protocols before and after freezing (e.g., hand inversion, vortex-mixing, centrifuging), storage temperature (falsely low albumin concentrations have been reported for samples stored at −20 °C [27–29]), sample pH (where urine alkalinization to pH >8.0 can prevent sample degradation during long-term frozen storage at −20 °C [30]), and type of assay (e.g., radioimmunoassay, immunoturbidimetric, enzyme-linked immunosorbent) [29]. As well, samples with higher albumin concentrations at baseline may be less affected by long-term storage [26, 27, 30-32]. For these reasons, consensus recommendations are that urine samples should be analyzed for ACR within 1 week of collection (stored between 2–8 °C) [31, 33].

In our study, we found that averaging ACR values from two consecutively collected first-morning urine samples reduced the required sample size by approximately 30 %, where the total number of participants needed to detect a difference of at least 15 % was 414 for single samples and 286 for the average of two samples. These calculations were based on log-transformed data using the standard deviation of the log-transformed percentage change in ACR [18]. Sample size estimates based on un-transformed data were twice as high as estimates based on log-transformed data. For a study where the primary outcome is the difference between two means with skewed distributions, the log transformation is preferred over other types of transformations for reasons of interpretability because unlike other transformations, log-transformed data can be back-transformed to provide an interpretable 95 % confidence interval for the difference in geometric means [34]. For example, taking the anti-log of the confidence limits provides the 95 % confidence interval for the ratio of two geometric means (where the difference between the logarithms of two geometric means equates to the logarithm of their ratio) [35]. Although the log transformation is preferred for reasons of interpretability, the suitability of the transformation should always be assessed by examining the shape of the distribution after transformation and the variance ratio between comparison groups [34].

The strengths of our study are that it evaluates a simple highly utilized readily available laboratory test and includes the use of multiple methods to reduce intra- and inter-individual variations of ACR. Because urine protein concentration can vary with physical activity, diurnal factors, diet, and hydration, we used multiple consecutive first-morning samples. To minimize the effects related to laboratory analysis (e.g., calibration, assays, reagents, and detection levels), all samples were analyzed at the same laboratory [36], and since urine protein is known to degrade at higher temperatures and over time, we ensured that samples were stored at 4 °C and analyzed on the same day of collection for our primary analysis, as recommended [31, 33]. The major limitation of this study is its small sample size, which may have resulted in an overestimation of ACR variability; others have shown that the effect of intra-individual ACR variability is minimized with larger sample sizes [6]. Further, the variability of ACR decline may vary with baseline ACR, CKD stage, and primary cause of chronic kidney disease; however, our study was not designed nor powered to examine these questions. As well, our findings are based on pilot data from a small clinic-based sample from a single center and thus may not be broadly generalizable. Although our home urine collection protocol worked well in this urban population (where all participants were residents of London, Ontario, and urine samples were picked up by hospital courier), including participants from rural areas would require different arrangements for sample pick up. As well, our study sample was 88.5 % male, which is notably higher than the typical 2:3 male-to-female ratio seen in chronic kidney disease populations at our center [37] and elsewhere [6, 30]. It is not clear whether this discrepancy is related to the study protocol or is simply a random effect of the small sample size.

## Conclusions

We successfully used a home urine collection protocol to obtain multiple consecutive first-morning urine samples in outpatients with chronic kidney disease. Variability in the percentage change in ACR was reduced when ACR from two consecutive first-morning urine samples was averaged (compared with single urine collections). No further reduction in sample variability was achieved with three consecutive samples. In clinical trials using percentage change in ACR as an endpoint, statistical efficiency may be improved by averaging ACR from two consecutive first-morning urine samples at both baseline and follow-up.
